# Cause or consequence? Understanding the role of cortisol in the increased inflammation observed in depression

**DOI:** 10.1016/j.coemr.2022.100356

**Published:** 2022-06

**Authors:** Nare Amasi-Hartoonian, Luca Sforzini, Annamaria Cattaneo, Carmine Maria Pariante

**Affiliations:** 1 Institute of Psychiatry, Psychology and Neuroscience, King's College London, Department of Psychological Medicine, London, UK; 2 Department of Pharmacological and Biomolecular Sciences, University of Milan, Milan, Italy; 3 Laboratory of Biological Psychiatry, IRCCS Istituto Centro San Giovanni di Dio Fatebenefratelli, Brescia, Italy; 4 National Institute for Health and Research Biomedical Research Centre at South London and Maudsley NHS Foundation Trust and King’s College London, UK

**Keywords:** Hypothalamic-pituitary-adrenal axis, inflammation, major depressive disorder, glucocorticoid resistance, cortisol, glucocorticoid receptor

## Abstract

Glucocorticoids such as cortisol are a class of steroid hormones that play an important role in co-ordinating the body’s response to stress. Elevated cortisol levels and increased inflammation have frequently been reported in patients with depression. The currently accepted “glucocorticoid resistance” model posits this increased inflammation as a consequence of reduced sensitivity to cortisol’s putative anti-inflammatory action. However, opposing evidence has accumulated that supports a more recent model, which instead proposes that cortisol possesses immune potentiating properties and may thus directly cause the increased inflammation seen in depression. Despite all of this, a clear explanation of the neuroendocrine mechanism that contributes to the development of depression is still lacking and thus requires further investigation in improved future studies.

## 2 Introduction

Increased inflammation and hypothalamic-pituitary-adrenal (HPA) axis alterations have been consistently associated with depression, or at least with a subgroup of individuals with depression (Pariante, 2017). It is widely known that the HPA axis is strictly implicated in inflammation. Its activation triggers the release of glucocorticoids, mainly cortisol in humans, which plays a crucial role in anti-inflammatory and immunosuppressive processes (Bellavance & Rivest, 2014). However, the clear relationships between the HPA axis and inflammation and depression and with one another have yet to be fully elucidated.

Initial findings regarding the neuroendocrine and biological correlates of major depressive disorder (MDD) consistently identified HPA axis hyperactivity and subsequent increased cortisol levels, which have also been observed in patients with treatment-resistant depression that is associated with greater severity. As a result, the “glucocorticoid resistance” theory emerged in the late 90s, which proposes that the glucocorticoid receptor (GR) is less sensitive to cortisol and does not bind as effectively, thus the regulation of the HPA axis through negative feedback inhibition becomes impaired, resulting in continued activation and production of the axis components (Pariante & Miller, 2001; Pariante & Lightman, 2008; Anacker et al., 2011). This diminished sensitivity of the GR is considered to be due to reduced GR function and expression that has been reported in depression by a large number of experimental, biological and molecular studies.

During the last 10 years, evidence started to accumulate not only from animal studies showing that repeat social defeat (a form of chronic stress) can induce glucocorticoid-resistant monocytes, enhanced neuroinflammatory signalling and depressive-like behaviours in animal models (Weber et al., 2017), but also from human studies that noted the coexistence of reduced GR function/expression or HPA axis hyperactivity and elevated inflammation in depressed patients (Pariante, 2017). Therefore, scientists naturally inferred that this single molecular mechanism of glucocorticoid resistance is the common factor that is related to both HPA axis function and immune activation. That is to say, immune cells, which express GR (Ménard et al., 2017), become less sensitive to cortisol’s ‘physiological anti-inflammatory’ action that may lead to the escape and hyperactivity of monocytes (and other immune cells), thus explaining the resulting increased inflammation that is observed. But despite all of this, the evidence is conflicting, as studies have reported findings that are in disagreement with the current or simplest version of the glucocorticoid resistance model.

## 3 Inflammation, HPA axis and glucocorticoids in MDD

Results from recent meta-analyses (Osimo et al., 2020; Osimo et al., 2019) and case-control studies (Chamberlain et al., 2019; Lynall et al., 2020; Cattaneo et al., 2020) have confirmed the presence of increased inflammation in depression, particularly in treatment-resistant patients, through elevated levels of inflammatory markers like the C-reactive protein and higher immune cell counts compared with healthy controls, even in treatment-responsive patients. Most importantly, the largest study to have corroborated this finding utilised data on approximately 27 thousand people with MDD from the UK Biobank and suggested the existence of a core biological association between depression and increased inflammation, since this association remains significant after adjusting for clinical and psychosocial confounding factors (Pitharouli et al., 2021). Several human and animal studies have shown that this association is most likely due to stress system activation, leading to the release of pro-inflammatory cytokines and activated immune cells like monocytes, which occur along with HPA axis hyperactivity and increased cortisol levels (Raison et al., 2006; Miller & Raison, 2016; Raison & Miller, 2003). Moreover, associations were found between specific inflammatory markers and different MDD symptoms (Kappelmann et al., 2021; Fried et al., 2020; Felger et al., 2020), and individuals exposed to interferon-α, a pro-inflammatory trigger, displayed depressive-like symptoms (Nettis et al., 2020; Russell et al., 2019). Hence, clinical trials have investigated the putative therapeutic benefits of anti-inflammatory treatment in inflamed MDD/depressed patients with promising results (Nettis et al., 2021; Ménard et al., 2017).

However in the 2000s, evidence opposing the glucocorticoid resistance model began to surface. For example, Munhoz and colleagues (2006) reported that, contrary to the anti-inflammatory effects of glucocorticoids via NF-κB inhibition, rats exposed to chronic unpredictable stress had elevated glucocorticoid levels, which resulted in increased NF-κB activation and pro-inflammatory gene expression induced by exposure to acute stress. Furthermore, pre-treatment with a GR antagonist weakened this effect, thus suggesting the putative immune potentiating properties of glucocorticoids. This pro-inflammatory action has also been observed in work conducted in our lab in human hippocampal progenitor cells that were treated with glucocorticoids prior to an inflammatory stimulus (Horowitz et al., 2020).

Moreover, investigating the biological outcomes of different types of stress in male mice failed to yield a consistent finding of HPA axis hyperactivity and increased inflammation, as researchers in our lab found that physical stress leads to hypercortisolaemia and reduced pro-inflammatory cytokine levels, while psychosocial stress leads to hypocortisolaemia and elevated pro-inflammatory cytokine levels (Du Preez et al., 2020). Furthermore, again in our lab, Perrin and colleagues (2019) failed to identify a strong positive correlation between glucocorticoid resistance and inflammation in their meta-analysis of studies examining both cytokine levels in depressed patients and measures of glucocorticoid resistance, including plasma cortisol levels, dexamethasone (synthetic glucocorticoid) suppression test, *GR* expression and *in vitro* assays of GR function. Similarly, Cattaneo et al. (2020) did not report a clear correlation between serum CRP inflammatory marker levels and glucocorticoid-related gene expression.

Therefore, this recent information has led the scientific community to postulate that the inflammation seen in depression may not solely be a *consequence* of glucocorticoid resistance and reduced GR signal but rather could be *caused* by the potential pro-inflammatory action of cortisol whose levels are aberrantly increased due to HPA axis hyperactivity.

## 4 The two models

**Figure 1:**
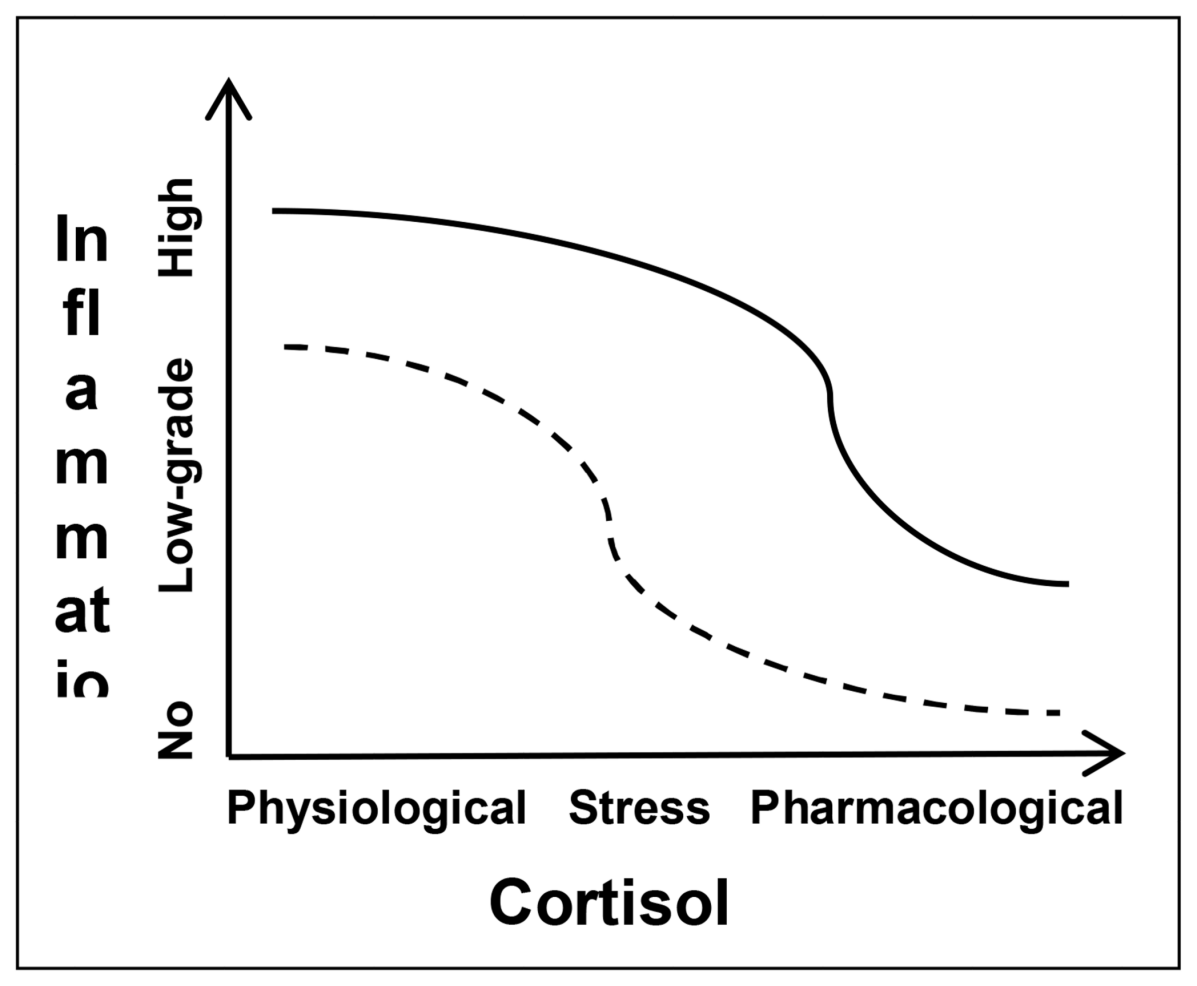
The glucocorticoid resistance model

The **“glucocorticoid resistance” model** was first proposed in the 1990s in the context of inflammatory diseases like asthma and inflammatory bowel disorders (Lamberts, 1996), and later became an established finding in psychiatry in the 2000s (Raison & Miller, 2003; Pariante & Miller, 2001). This phenomenon is the current consensus theory for HPA axis hyperactivity and the accompanied increased inflammation that are observed in depression. On the basis of this model, the **physiological anti-inflammatory action** of cortisol in humans increases as its concentration rises, that can range from *physiological levels* (during the day), to *stress levels* (those seen in depressed patients or that is induced experimentally) and to *pharmacological levels* (that is achieved with a large hydrocortisone or comparable synthetic glucocorticoid administration). A visual representation of this model is depicted in Figure 1 where despite the higher cortisol levels in depressed patients (**straight line**) compared to healthy controls (**dotted line**), their immune cells are resistant to cortisol’s aforementioned anti-inflammatory action, thus inflammation is less inhibited in these individuals at different concentrations of cortisol than controls, hence resulting in the increased inflammation that is seen in depression. The scientific literature investigating this resistance to cortisol points to abnormalities involving the GR, which under normal conditions has a low affinity for cortisol and therefore requires high glucocorticoid concentrations to be fully activated (Anacker et al., 2011). Reviews and primary research have reported reduced function and/or expression of the GR in the immune cells of depressed patients, particularly those that are inflamed (Pariante & Miller, 2001; Anacker et al., 2011; Pariante & Lightman, 2008; Mariani et al., 2021; Cattaneo et al., 2020; Cattaneo 2013). This is considered to be influenced by gene-environment interactions at *FKBP5*, a negative regulator of GR function, whereby early life adversity (even *in utero*) and *FKBP5* risk alleles can lead to or exacerbate epigenetic alterations in this gene, thus increasing MDD risk (Mourtzi et al., 2021; Lin and Tsai, 2019; Matosin et al., 2018) and potentially promoting NF-κB-driven peripheral inflammation (Zannas et al., 2019). Other evidence supporting this model includes the reports of glucocorticoid resistance, inflammation and depressive-like behaviours in the aforementioned repeated social defeat animal models of depression (Weber et al., 2017) and a study showing that administration of dexamethasone (a synthetic glucocorticoid and GR agonist) leads to reduced GR target gene expression in patients with depressive disorders and mouse models (Arloth et al., 2015).

However, despite the existing findings in favour of this model, the previously mentioned meta-analysis only reported modest support for the association between glucocorticoid resistance and cytokine-mediated inflammation in depressed patients compared to controls (Perrin et al., 2019). In addition, the authors noted not only the limited number of included articles and study subjects for which information on both glucocorticoid resistance and inflammation was collected, but also that most individual studies had utilised only a single method to quantify glucocorticoid resistance.

**Figure 2:**
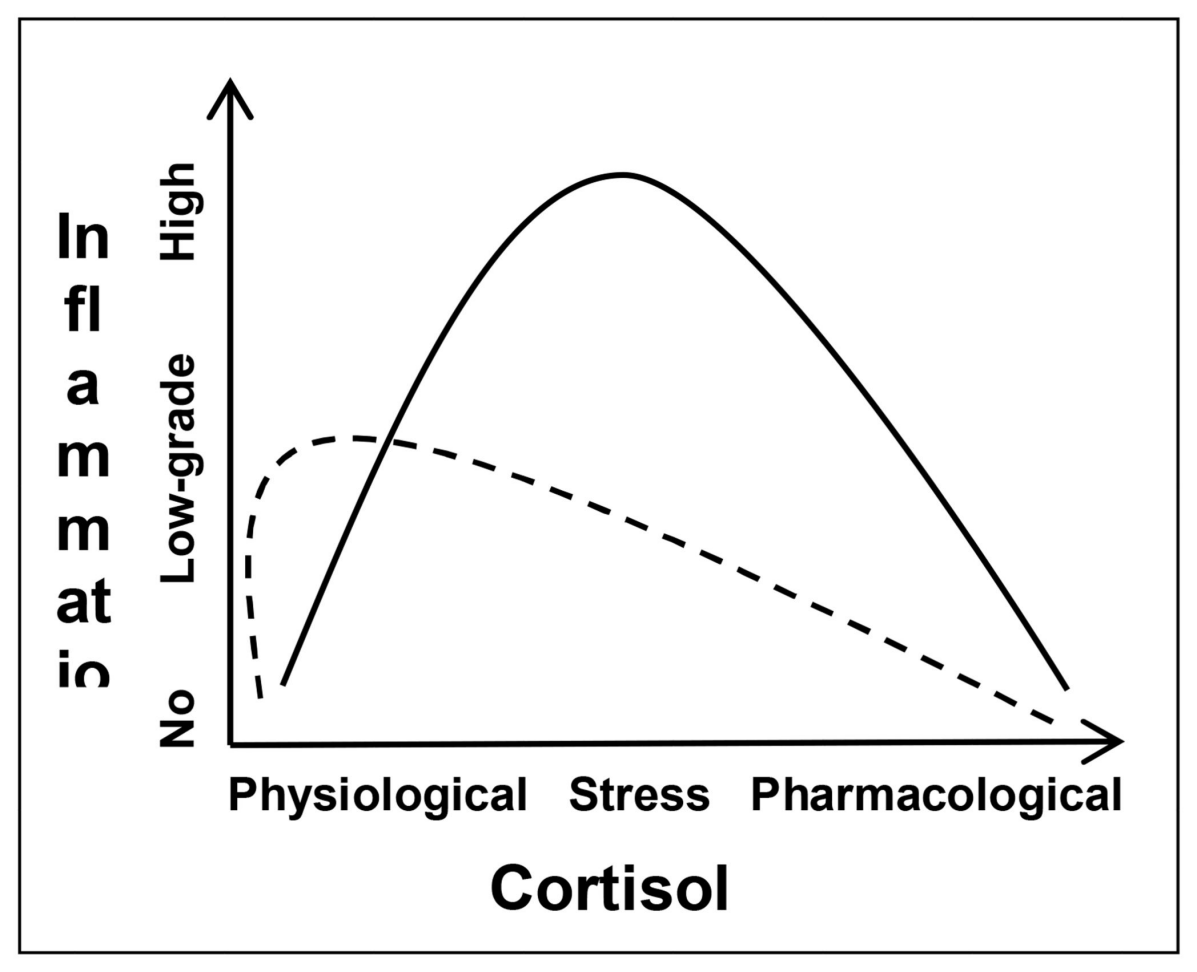
The pro-inflammatory cortisol model

The accumulation of more recent evidence against the current model has resulted in a conceptual shift; providing an alternative explanation to the notions outlined in the first review published on the relevance of glucocorticoid resistance in depression (Pariante & Miller, 2001). This newer **“pro-inflammatory cortisol” model** that has emerged, proposes the idea that glucocorticoids may possess **pro-inflammatory properties** during stress, and so the high cortisol levels, typically observed in depressed patients, may be the *cause* of the elevated inflammation in MDD, instead of just a *consequence* of glucocorticoid resistance. This phenomenon is supported by findings from animal research demonstrating that increased concentrations of corticosterone (the primary glucocorticoid in rodents) induced by chronic unpredictable stress or achieved through glucocorticoid administration/manipulation resulted in enhanced inflammation in response to acute stress (Munhoz et al., 2006, 2010). Furthermore, Niraula and colleagues (2018) found that administration of metyrapone (a glucocorticoid synthesis inhibitor) and surgical removal of the adrenal glands (where glucocorticoids are synthesised) to inhibit corticosterone production, led to the prevention of neuroinflammatory signalling and inflammatory monocyte release into circulation when exposed to repeated social defeat stress, indicating that corticosterone increases inflammation in this model. In healthy human subjects, exposure to stress-associated concentrations of cortisol for 6 hours via hydrocortisone (intravenous cortisol) administration, elicited a pro-inflammatory response, including a significant increase in IL-6 cytokine levels, following an inflammatory stimulus (Yeager et al., 2016, 2011). Similar findings were reported by Horowitz et al. (2020) in our lab, who investigated the effect of dexamethasone or cortisol treatment in human hippocampal progenitor cells prior to an inflammatory stimulus, which resulted in the increased expression of several innate immune genes. Moreover, these studies interestingly observed that this pro-inflammatory effect was maximal at intermediate (stress-relevant) cortisol levels, but not at high (pharmacological) or low (physiological) concentrations, and when there was a delay or rest period of 24 hours before the inflammatory stimulus. This model has not yet been tested in human patients with depression. As displayed in Figure 2, this alternative model postulates that cortisol possesses a pro-inflammatory action and so the elevated stress-levels of this hormone that are found in depressed patients (**straight line**) compared to healthy controls (**dotted line**) is in fact the reason for the increased inflammation that is present in these individuals, and not just merely resulting from glucocorticoid resistance.

## 5 Epigenetics and cell/tissue type

The role of epigenetic mechanisms has been well investigated in relation to the “glucocorticoid resistance” model, with several studies reporting the link between early life adversity/trauma, depression or stress-related conditions and the hypomethylation of the aforementioned *FKBP5* gene or hypermethylation of the *NR3C1* (GR) gene promoter, which leads to decreased GR activity or decreased GR mRNA and protein expression (Mourtzi et al., 2021; Farrell and O’Keane, 2016; Spies et al., 2021). This reduced *FKBP5* methylation was also associated with promoting inflammation (Palma-Gudiel et al., 2021; Zannas et al., 2019), although more studies including this measure are required, as well as further research to clarify inconsistent findings (Lin and Tsai, 2019; Farrell and O’Keane, 2016). Moreover, animal and cell culture studies found that histone deacetylation correlated with reduced transcription factor binding to the GR promoter and depressive behaviours (Farrell and O’Keane, 2016), in addition to miRNA overexpression resulting in GR downregulation (Jung et al. 2015; Vreugdenhil et al., 2009).

The contribution of epigenetics within the context of the “pro-inflammatory cortisol” model has not yet been explored. However, it is known that the GR interacts with coactivators that possess histone acetyltransferase activity to regulate immune gene expression and the suggested mechanisms for the immune-potentiating effects of glucocorticoids involves inducing the expression of genes with a pro-inflammatory function like *TLR2*, partly through GR interaction with NF-κB response elements (Liberman et al., 2018; Desmet & De Bosscher, 2017; Xavier et al., 2016).

It is also considered that glucocorticoids can exert contrasting actions depending on the cell or tissue type, with pro-inflammatory effects reportedly demonstrated in dendritic cells or the brain and anti-inflammatory effects in peripheral immune cells like neutrophils (Liberman et al., 2018; Xavier et al., 2016). Although, both of these effects have been observed within the same cell type (Cruz-Topete & Cidlowski, 2015; Xavier et al., 2016).

## 6 Future directions

In spite of this rationale, it is important to note that the interplay between the endocrine and immune systems is complex and paradoxical (Perrin et al., 2019), since cortisol is not only implicated in increasing inflammation, but it can also bind the GR and repress the expression of genes encoding pro-inflammatory cytokines (Anacker et al., 2011). Moreover, this relationship is bidirectional as pro-inflammatory cytokines themselves can inhibit GR function (Pariante et al., 1999) through activation of mitogen-activated protein kinases like p38 (Pariante & Lightman, 2008) and JNK (Zhang et al., 2020) thus in turn contributing to glucocorticoid resistance resulting in a feed-forward inflammatory cascade (Raison & Miller, 2003).

A potential explanation of cortisol’s biphasic effects on the immune system (that seem to be not only time- and dose-dependent but also reliant on the GR) may be that stress-concentrations of glucocorticoids prime the innate immune system resulting in the existence of a certain-level of intrinsic inflammation that leads to the exacerbating consequences stated above that are seen in depression (Perrin et al., 2019; Horowitz et al., 2020; Yeager et al., 2011). Therefore, it can be speculated that the proposed models may occur simultaneously.

However, considering also the limitations of previous clinical studies, including small sample sizes, there is still a need for additional large mechanistic studies that specifically investigate cortisol’s GR-mediated effects on cytokine production in human depression subjects through utilising more comprehensive measures in order to disentangle this relationship.
